# Does methotrexate influence COVID-19 infection? Case series and mechanistic data

**DOI:** 10.1186/s13075-021-02464-4

**Published:** 2021-06-10

**Authors:** Fabian Schälter, Kerstin Dürholz, Laura Bucci, Gerd Burmester, Roberto Caporali, Camille Figuereido, Jaime Fogagnolo Cobra, Bernhard Manger, Mario M. Zaiss, Georg Schett

**Affiliations:** 1 Department of Internal Medicine 3, Friedrich-Alexander University (FAU) Erlangen-Nürnberg and Universitätsklinikum Erlangen, Erlangen, Germany; 2 Deutsches Zentrum fuer Immuntherapie (DZI), Friedrich-Alexander University (FAU) Erlangen-Nürnberg and Universitätsklinikum Erlangen, Erlangen, Germany; 3 Department of Rheumatology and Clinical Immunology, Charité, Berlin, Germany; 4 Department of Clinical Sciences and Community Health, Research Center for Adult and Pediatric Rheumatic Diseases, University of Milan, G. Pini Hospital, Milan, Italy; 5 Cobra Clinic of Rheumatology and Research Center, São Paulo, Brazil; 6 Department of Rheumatology, University of Sao Paulo, São Paulo, Brazil

**Keywords:** Methotrexate, Coronavirus disease 19, Infection

## Abstract

**Background:**

To investigate whether methotrexate treatment may affect the susceptibility to infection with severe acute respiratory syndrome coronavirus 2 (SARS-CoV-2).

**Methods:**

Clinical assessment of symptoms, SARS-CoV-2 RNA, and anti-SARS-CoV-2 IgG in an initial case series of four families and confirmatory case series of seven families, within which one family member developed coronavirus disease 19 (COVID-19) and exposed another family member receiving methotrexate treatment; experimental part with methotrexate treatment of mice and organoids followed by the assessment of mRNA and protein expression of the SARS-CoV-2 receptor angiotensin-converting enzyme (ACE)-2.

**Results:**

In the initial case series, three of four women on a joint ski trip developed COVID-19, while the fourth woman, under treatment with methotrexate, remained virus-free. Two of the three diseased women infected their husbands, while the third husband treated with methotrexate remained virus-free. In addition, 7 other families were identified in a follow-up case series, in which one member developed COVID-19, while the other, receiving methotrexate, remained healthy. Experimentally, when mice were treated with methotrexate, ACE2 expression significantly decreased in the lung, in the intestinal epithelium, and in intestinal organoids.

**Conclusion:**

These clinical and experimental data indicate that methotrexate has certain protective effects on SARS-CoV-2 infection via downregulating ACE2.

## Background

Severe acute respiratory syndrome coronavirus 2 (SARS-CoV-2) enters epithelial and other cells through binding to angiotensin-converting enzyme 2 (ACE2) expressed on various epithelial cells including those of the lungs and the gut [1]. SARS-CoV-2 entry into human cells triggers cell damage associated with a robust and sometimes overshooting inflammatory responses in the affected organs and the development of coronavirus disease 19 (COVID-19 [2].

The level of ACE expression by lung epithelia may influence the susceptibility to SARS-CoV-2 infection. For instance, low ACE2 expression in young mammals can potentially explain the overall low susceptibility of children to COVID-19 and other zoonotic coronaviruses [3]. Furthermore, smoking as well as high salt diet have shown to increase ACE2 expression, which may explain the higher risk for COVID-19 in smokers and those with hypertension, respectively [4, 5]. In addition, administration of recombinant soluble ACE2 saturates cellular surface ACE2 outcompetes viral binding sites and hence is effective in preventing SARS-CoV-2 infection of human tissue organoids [6].

To date, little is known on whether anti-inflammatory drugs used for the treatment of immune-mediated chronic inflammatory diseases (IMIDs), including arthritis, could influence ACE2 expression and affect susceptibility to SARS-CoV-2 infection. Herein, we report on case series of families, in which one family member developed COVID-19, while the other family member, taking methotrexate (MTX), remained virus-free and healthy despite substantial viral exposure.

## Methods

### Ethical approval

Ethical approval (#157_20 B) to conduct this analysis was granted by the institutional review board (IRB) of the University Clinic of Erlangen. Written informed consent was obtained from the study participants.

### Participants

Data were obtained from [1] an initial patient group of 4 women performing a ski holiday at Ischgl (Austria) and their respective husbands and 7 confirmatory cases from Italy [2], Brazil [4] and Germany [1] comprising families with one member infected with COVID-19 and the other member taking methotrexate staying healthy. In the initial patient group, methotrexate was used for treatment of psoriasis [1] and rheumatoid arthritis [1]. In the confirmatory cases, methotrexate was used for treatment of rheumatoid arthritis [4] and psoriatic arthritis/spondyloarthritis [3]. Doses of methotrexate were between 10 and 20 mg/week.

### Symptoms

Participants were asked for their clinical symptoms (cough, rhinitis, throat pain, fever, headache, fatigue, musculoskeletal pain, anosmia, shortness of breath, and diarrhea) according to a questionnaire that was used for a field study on anti-SARS-CoV-2 IgG [7]. The observation period in the initial and confirmatory cases was 1 month.

### SARS-CoV-2 RNA test

RNA quantification was down from mucosal swabs using Cobas SARS-CoV-2 real-time RT-PCR Test (Roche, Basel, CH).

### Anti-SARS-CoV-2 antibody testing

Serum samples were taken between March 18 and April 30 for anti-SARS-CoV-2 IgG tests. Immunoglobulin G (IgG) antibodies against the S1 domain of the spike protein of SARS-CoV-2 were tested by the recent CE version (April 2020) of the commercial enzyme-linked immunosorbent assay from Euroimmun (Lübeck, Germany) using the EUROIMMUN Analyzer I platform according to the manufacturer’s protocol and as described previously [7]. Optical density was determined at 450 nm with reference wavelength at 630 nm. A cut-off of ≥0.8 (OD450nm) was considered as positive. Assays were performed in line with the guidelines of the German Medical Association (RiliBAK) with stipulated internal and external quality controls.

### Mice

Female, C57Bl/6 N mice were maintained under specific pathogen-free conditions at the Präklinisches Experimentelles Tierzentrum (PETZ) Erlangen, Germany, and approved by the local ethics authorities of the Regierung of Unterfranken (#55.2-2532.1-59/14). Nine-week-old mice were acclimated for 1 week, followed by a 3-week co-housing period before i.p. treatment with 2 mg/kg mouse methotrexate (*n* = 6) or vehicle (PBS; *n* = 6) started for three consecutive days before analysis. Two independent experiments were performed.

### Organoids

Intestinal organoids were generated as previously described [8]. In short, the small intestine of wild-type C57Bl/6 J was removed, longitudinally opened, and villi were scraped out. Isolated intestinal crypts were resuspended in 25 μl/well growth factor reduced Matrigel (Corning, NY, USA). Organoids were cultured in the incubator for at least 7 days before any experiments were performed. Medium was changed every 2 to 3 days and organoids were split once per week. Organoids were stimulated with 0.25, 0.5, or 1 mM methotrexate for 6 h before analysis.

### Western blot

Organoids were washed two times with PBS and then subsequently lysed with RIPA buffer. Protein extract were separated on a 4–12% SDS-polyacrylamide gel, transferred to a nitrocellulose membrane, and stained with antibodies against murine ACE2 (R&D systems). An antibody against murine β-actin (Abcam) was used as loading control. Densitometry analysis was performed using ImageJ.

### Histology

Lung and intestinal tissue were fixed in 4% formalin. After deparaffinization, rehydration, and blocking, paraffin sections were stained using a primary antibody against murine ACE2 (R&D systems). Slides have been developed using a DAB staining solution kit after incubation with a HRP-conjugated anti-goat secondary antibody. Quantification of stained area per slide was performed using ImageJ.

### Statistical analysis

Data are expressed as mean ± s.d. Analysis was performed using Student’s *t* test, single comparison, or analysis of variance (ANOVA) test for multiple comparisons (two-way ANOVA followed by Tukey’s or Bonferroni’s multiple comparisons test). All experiments were conducted at least two times. *P* values of 0.05 were considered significant and are shown as *p* < 0.05 (*), *p* < 0.01 (**), or *p* < 0.001 (***). Graph generation and statistical analyses were performed using the Prism version 8 software (GraphPad, La Jolla, CA).

## Results

### Initial case series

We were first contacted by a group of four Caucasian women, aged between 53 and 62 years, who lived in different households in Germany and had traveled for ski holidays to Ischgl (Tyrol, Austria) from Feb 29 to March 4, 2020. They shared the car for going there (approximately 4-h ride) and stayed in the same room the entirety of their holiday. They regularly visited ski huts and restaurants for lunch and dinner. All of them were in good physical condition and none had concomitant diseases with the only exception that one of the four was on stable methotrexate treatment (15 mg/week) for rheumatoid arthritis. Her arthritis was in remission.

**Fig. 1:**
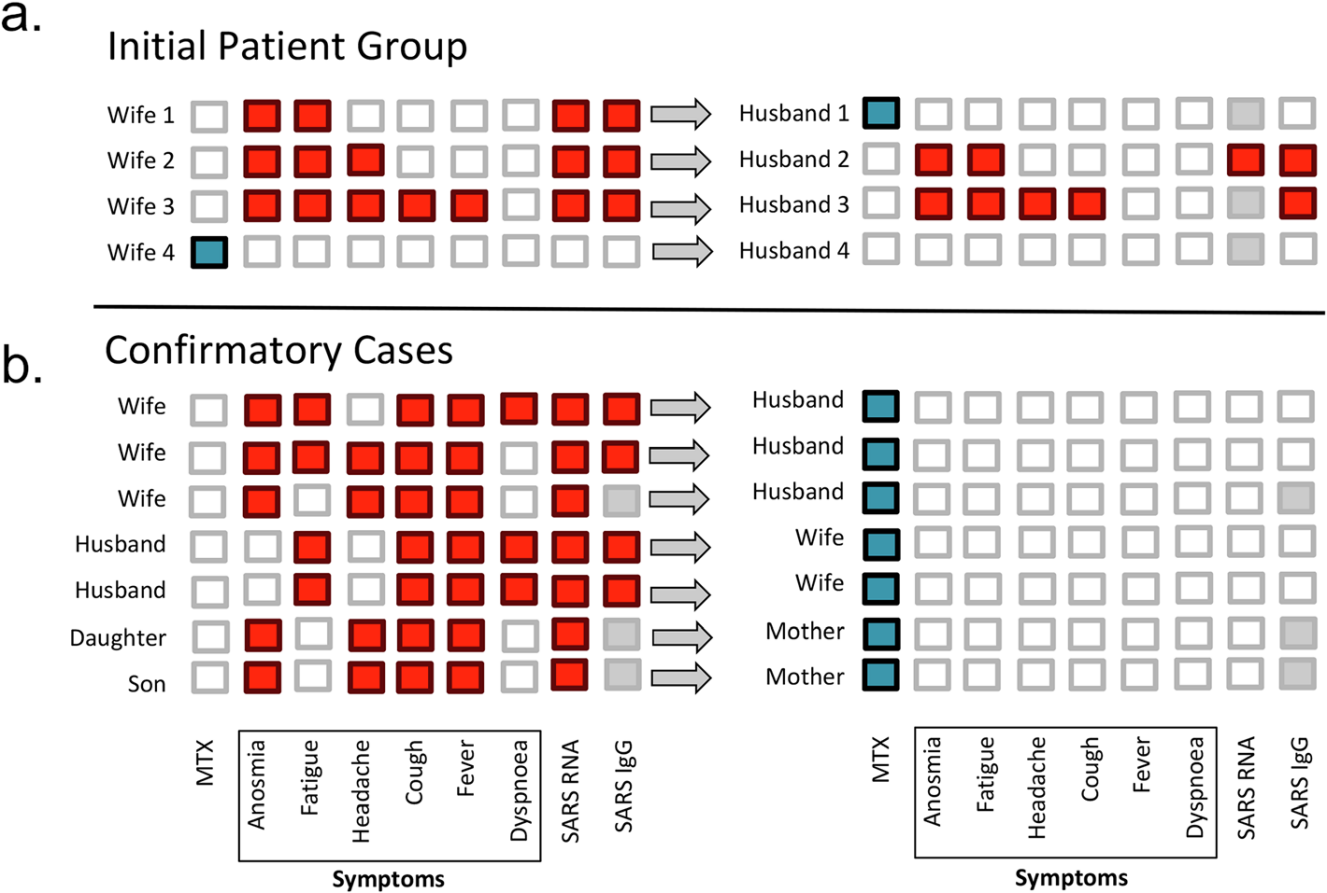
MTX inhibits SARS-CoV-2 infection in highly exposed individuals. **a** Initial patient group of four women and their respective husbands. **b** Confirmatory cases. Symptoms related to COVID-19 and results of mucosal swabs for SARS-CoV-2 RNA and SARS-CoV-2 IgG antibody serum testing are shown. Red squares: positive or present; white squares: negative or absent; gray squares: not tested. Methotrexate use indicated by blue squares

All four remained healthy during the holidays, but 2 days after their return, three developed infectious symptoms including fever, cough, headache, and fatigue (Fig. 1a). Anosmia was reported by all of them. The fourth woman taking methotrexate developed no symptoms. Five days after the start of symptoms, RNA tests for SARS-CoV-2 were taken from mucosal swabs and the three women with symptoms were all tested positive. The woman taking methotrexate showed a negative SARS-CoV-2 RNA test. Three weeks later, serums of all four women were tested for anti-SARS-CoV-2 IgG (Euroimmun ELISA). The three symptomatic women, who had previously tested positive for SARS-CoV-2 RNA, were also highly positive for anti- SARS-CoV-2 IgG, while the one on methotrexate remained negative. The three infected women recovered within 2 weeks without complications.

All women lived with their husbands in the respective households. No other family members lived in these households. Two of the three husbands of the infected women developed anosmia, fatigue, headache, and cough within 7 days after return of their wives. The third husband, living with one of the infected women, did not develop any symptoms. He was on stable treatment with methotrexate (20 mg/week) for psoriasis. Mucosal swab was done, in which that taken from one of the two infected husbands confirmed for COVID-19 RNA. Moreover, all four husbands (including the one of the healthy woman) were tested for anti-SARS-CoV-2 IgG. The two symptomatic husbands were highly positive for anti-SARS-CoV-2 IgG, while the asymptomatic husband on methotrexate as well as the husband from the non-infected woman remained IgG negative. Both husbands with COVID-19 recovered from the infection without complications.

### Confirmatory cases

Based on these observations we searched for additional families, in which one of the family members developed RNA- confirmed COVID-19, while the other family member taking MTX remained healthy. We identified 7 additional couples or parents/children, in whom the person taking methotrexate remained healthy and SARS-CoV-2 RNA negative despite living in the same household and in close contact with a COVID-19 case. Four of the seven cases received MTX for rheumatoid arthritis, while the other three individuals had psoriatic arthritis or peripheral spondyloarthritis. Details of the disease symptoms are shown in Fig. 1b.

### Mechanistic studies

**Fig. 2:**
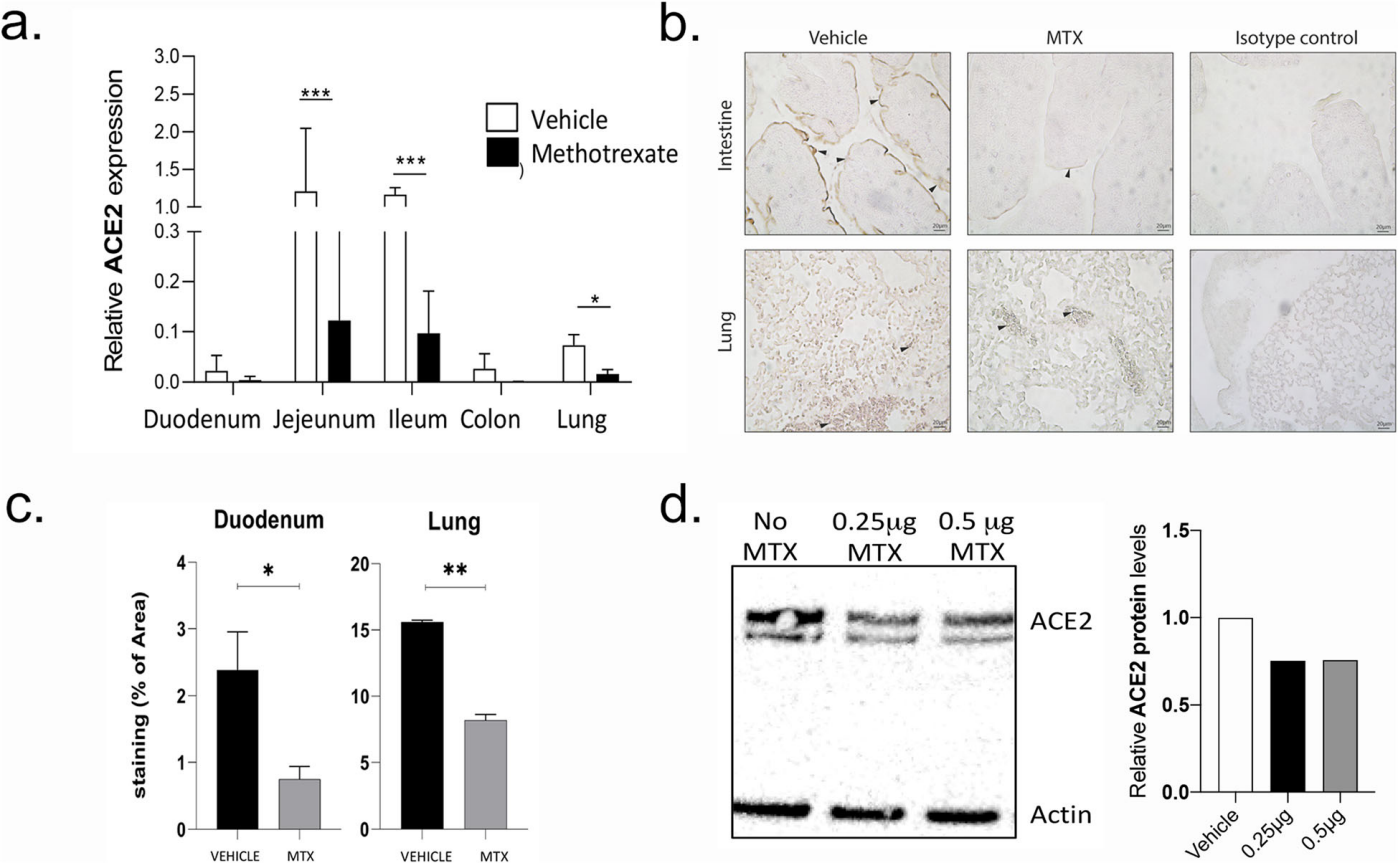
MTX down-regulates ACE2 expression. **a** Real-time PCR showing quantification of mRNA for ACE2 in the various segments off the intestine and the lung in mice treated with methotrexate (MTX; *N* = 3 per group per experiment; data summarized from two independent experiments). **b** Representative immune histochemistry staining for ACE2 (brown color, arrowheads) in the intestine and the lung of mice treated with methotrexate or vehicle for 3 days. Isotype control antibody staining is also shown. **c** Quantification of ACE2 expression in immune histochemistry (*N* = 3 per group per experiment; data summarized from two independent experiments). **d** Representative immunoblot for ACE2 and actin (control) in intestinal organoids treated with two doses of methotrexate including densitometric quantification

As these observations suggested that MTX treatment is associated with at least some level of protection from COVID-19, we hypothesized that MTX may affect expression of ACE2, the receptor of the SARS-CoV-2 [1]. We therefore treated mice with MTX for 3 days and then tested the RNA expression of ACE2 in the lungs and in the intestine by RT-PCR. In addition, we tested ACE2 protein expression by immunohistochemistry in both organs. MTX treatment led to a significant downregulation of ACE2 mRNA (Fig. 2a) and protein expression (Fig. 2b and c) in the lungs and in the intestine. Furthermore, when intestinal organoids were treated with methotrexate at concentration reflecting human plasma levels in rheumatoid arthritis [9], ACE2 protein expression was down regulated (Fig. 2d).

## Discussion

MTX is a widely and long-used drug to effectively treat IMIDs, in particular rheumatoid and psoriatic arthritis [10]. While doses of MTX used for the treatment of arthritis have potent ant-inflammatory properties, they are not generally immune suppressive and have not been shown to increase the risk of viral infections. MTX induces the generation of adenosine, which has anti-inflammatory properties and likely mediated the inhibitory effect of MTX on arthritis [11, 12]. It is interesting that adenosine inhibits lung inflammation in the context of LPS challenge and also inhibits NETosis and thrombosis [13, 14], which are hallmarks of COVID-19 [15]. Previous data have also suggested a link between ACE2 expression and adenosine metabolism but have not described the regulation of ACE2 by MTX [16].

Our data show that MTX-treated patients can remain healthy despite long and close contact to SARS-CoV-2-infected individuals, which is remarkable. Since MTX mitigates but not abrogates the expression of ACE2, the protective effect of MTX is definitely not complete and may be overridden by other factors such as high viral load or the aforementioned inducers of ACE2 expression. Cohorts investigating IMID patients with COVID-19 have not shown a difference in the prevalence of MTX between mild (non-hospitalized) and severe (hospitalized) patients [17–19]. These observations suggest that MTX may not inhibit the hyper-inflammatory response in the patients infected by SARS-CoV-2. In contrast, our data support the role of MTX in viral entry through inhibition of ACE2 expression, which is different from the regulation of SARS-CoV-2-induced host response. The observation that the MTX-treated patients of the two case series remained RNA-negative underlines this concept.

A limitation of this study is that we cannot conclude from the case series and from the experimental data that methotrexate indeed lowers the risk for COVID-19. The study has not been designed to answer this question, which can only be addressed in larger prospective cohorts. Furthermore, murine data have certain limitations since mouse ACE2 bind less efficiently than human ACE2 to SARS viruses [20]. Nonetheless, the data open doors to study such concept and to consider that anti-inflammatory drugs may not only influence the course of SARS-CoV-2 infection via modulation of inflammatory responses but also susceptibility to the infection via influencing virus entry. In addition, our data extend the concept that anti-inflammatory drugs used to treat diseases like rheumatoid arthritis are not broadly immune suppressive and may dampen rather than increase COVID-19 risk. In fact, several inflammatory pathways of the host that are induced by SARS-CoV-2 are shared by IMIDs such as arthritis [21]. In support, most studies so far showed that IMIDs treated with inhibitors of pro-inflammatory cytokines have no evidence for a more severe course of Covid-19 and with some treatments, e.g., TNF inhibitors, an even milder case of COVID-19 has been observed [16–18].

## Conclusion

Taken together these clinical and functional data indicate that MTX may have a certain protective effect on SARS-CoV-2 infection via downregulation of the expression of the viral receptor ACE2. However, to better define the inhibitory effect of methotrexate on the development of COVID-19, larger data sets including registry data and population-based data in high prevalence areas are needed.

## Data Availability

The datasets used and/or analyzed during the current study are available from the corresponding author on reasonable request.

## Abbreviations

ACE2: Angiotensin-converting enzyme 2
COVID-19: Coronavirus disease 19
IMIDs: Immune-mediated chronic inflammatory diseases
MTX: Methotrexate
SARS-CoV-2: Severe acute respiratory syndrome coronavirus 2
